# Looking through the Ice: Cold-Adapted Vision in Antarctic Icefish

**DOI:** 10.1093/molbev/msad058

**Published:** 2023-04-13

**Authors:** Casey McGrath

Antarctica may seem like a desolate place, but it is home to some of the most unique lifeforms on the planet. Despite the fact that land temperatures average around −60 °C and ocean temperatures hover near the freezing point of saltwater (−1.9 °C), a number of species thrive in this frigid habitat. Antarctic icefishes (Cryonotothenioidea) are a prime example, exhibiting remarkable adaptations that allow them to survive in the icy waters surrounding the continent. For example, these fish have evolved special “antifreeze” glycoproteins that prevent the formation of ice in their cells. Some icefishes are “white-blooded” due to no longer making hemoglobin, and some have lost the inducible heat shock response, a nearly universal molecular response to high temperatures. Adding to this repertoire of changes, a recent study published in *Molecular Biology and Evolution* reveals the genetic mechanisms by which the visual systems of Antarctic icefishes have adapted to both the extreme cold and the unique lighting conditions under Antarctic sea ice (Castiglione et al. 2023).

A team of researchers, led by Gianni Castiglione (now at Vanderbilt University) and Belinda Chang (University of Toronto), set out to explore the impact of subzero temperatures on the function and evolution of the Antarctic icefish visual system. The authors focused on rhodopsin, a temperature-sensitive protein involved in vision under dim–light conditions. As noted by Castiglione, a key role for rhodopsin in cold adaptation was suggested by their previous research. “We had previously found cold adaptation in the rhodopsins of high-altitude catfishes from the Andes mountains, and this spurred us into investigating cold adaptation in rhodopsins from the Antarctic icefishes.”

Indeed, the authors observed evidence of positive selection and accelerated rates of evolution in rhodopsins among Antarctic icefishes. Taking a closer look at the specific sites identified as candidates for positive selection, Castiglione and coauthors found two amino acid variants that were absent from other vertebrates. These changes are predicted to have occurred during two key periods in Antarctic icefish history: the evolution of antifreeze glycoproteins and the onset of freezing polar conditions. This timing suggests that these variants were associated with icefish adaptation and speciation in response to climatic events.

To confirm the functional effects of these two amino acid variants, the researchers performed in vitro assays in which they created versions of rhodopsin containing each variant of interest. Both amino acid variants affected rhodopsin's kinetic profile, lowering the activation energy required for return to a “dark” conformation and likely compensating for a cold-induced decrease in rhodopsin's kinetic rate. In addition, one of the amino acid changes resulted in a shift in rhodopsin's light absorbance toward longer wavelengths. This dual functional change came as a surprise to Castiglione and his coauthors. “We were surprised to see that icefish rhodopsin has evolved mutations that can alter both the kinetics and absorbance of rhodopsin simultaneously. We predict that this allows the icefish to adapt their vision to red-shifted wavelengths under sea ice and to cold temperatures through very few mutations.”

Interestingly, the amino acid changes observed in the Antarctic icefishes were distinct from those conferring cold adaptation in the high-altitude catfishes previously studied by the team, suggesting multiple pathways to adaptation in this protein. To continue this line of study, Castiglione and his colleagues hope to investigate cold adaptation in the rhodopsins of other cold-dwelling fish lineages, including Arctic fishes. “Arctic fishes share many of the cold-adapted phenotypes found in the Antarctic icefishes, such as antifreeze proteins. However, this convergent evolution appears to have been accomplished through divergent molecular mechanisms. We suspect this may be the case in rhodopsin as well.”

Unfortunately, acquiring the data needed to conduct such an analysis may prove difficult. “A major obstacle to our research is the difficulty of collecting fishes from Antarctic and Arctic waters,” says Castiglione, “which limits us to publicly available datasets.” This task may become even more challenging in the future as these cold-adapted fish are increasingly affected by warming global temperatures. As Castiglione points out, “Climate change may alter the adaptive landscape of icefishes in the very near future, as sea ice continues to melt, forcing the icefish to very likely find themselves at an evolutionary “mismatch” between their environment and their genetics.”
